# User Authentication in Smartphones for Telehealth

**DOI:** 10.5195/ijt.2017.6226

**Published:** 2017-11-20

**Authors:** KATHERINE A. SMITH, LEMING ZHOU, VALERIE J. M. WATZLAF

**Affiliations:** 1 DEPARTMENT OF SURGERY, UNIVERSITY OF PITTSBURGH, THOMAS E. STARZL TRANSPLANTATION INSTITUTE, PITTSBURGH, PA, USA; 2 DEPARTMENT OF HEALTH INFORMATION MANAGEMENT, SCHOOL OF HEALTH AND REHABILITATION SCIENCES, UNIVERSITY OF PITTSBURGH, PITTSBURGH, PA, USA

**Keywords:** Authentication, Biometrics, HIPAA, Mobile security, Telehealth

## Abstract

Many functions previously conducted on desktop computers are now performed on smartphones. Smartphones provide convenience, portability, and connectivity. When smartphones are used in the conduct of telehealth, sensitive data is invariably accessed, rendering the devices in need of user authentication to ensure data protection. User authentication of smartphones can help mitigate potential Health Insurance Portability and Accountability Act (HIPAA) breaches and keep sensitive patient information protected, while also facilitating the convenience of smartphones within everyday life and healthcare. This paper presents and examines several types of authentication methods available to smartphone users to help ensure security of sensitive data from attackers. The applications of these authentication methods in telehealth are discussed.

In 1973, Dr. Martin Cooper made the first call on a cellular phone (Thacker & Wilson, 2015). The phone was a large, bulky device that weighed about two and half pounds and measured 11 inches tall (Anjarwalla, 2010). It wasn’t until 1983 that Dr. Cooper’s cell phone became commercially available to the public (Thacker & Wilson, 2015). Since the development of the first cell phone, incredible advancements have been made to cellular devices. From large and bulky to small and sleek, cell phones, especially smartphones, became used less for phone calls and more for work and entertainment. Smartphones can now take and store pictures and videos; sense the environment; track physical activities; play games, check email accounts; visit websites, send text messages, and accomplish many other functionalities. Because of the great capabilities of smartphones, they have become an essential part of our daily life.

In November 2016, 95% of Americans owned a cell phone and 77% owned a smartphone -- a drastic increase from only 35% in 2011 (Pew Research Center, 2017). As smartphone users have grown, so have the numbers and use of applications on the mobile device. Professionals in various fields, including healthcare organizations, use their smartphones within their workplaces. Per a survey of 3,800 physicians, 83% own at least one mobile device and 25% use both smartphones and tablets within their clinical practice (Modahl, 2011). Eighty one percent (81%) of these physicians use their personal mobile devices to access patient records (Barrett, 2011), which raises grave concerns about information security, since patient health records are highly sensitive. A recent survey showed that 54% of smartphone users connect to Wi-Fi networks that have the potential to be insecure, while 20% of these same users access sensitive information, such as mobile banking, via insecure (public or shared) Wi-Fi networks (Olmstead & Smith, 2017). As the uses increase and more sensitive information is accessed via mobile phones, there is a growing need for users to be conscious of their mobile security.

Smartphones have become tremendously popular within the healthcare field for both providers and patients. Specific to telehealth, use of mobile health (mHealth) apps for telehealth services has expanded in recent years. A plethora of mHealth apps are available to download to smartphones that can help patients be more aware and in control of their healthcare. For example, some applications give the patient the ability to take and store photographs of a skin lesion to monitor potential progression (Kassianos, Emery, Murchie, & Walter, 2015). Some apps remind the patient to take a follow-up photograph for comparison, while other apps allow the patient to submit photographs to a physician for review (Kassianos, Emery, Murchie, & Walter, 2015, Parmanto, Pramana, Yu, Fairman, Dicianno, & McCue, 2013). These types of apps have great potential to help identify skin malignancies faster than if the patient were to schedule an office visit; however, users must also investigate how the app is storing and transmitting this collected information. For instance, some apps may store the information on the smartphone without sufficient protection, and some may forward the collected patient data to a cloud server located outside the United States without permission from the users.

A recent study sought respondents’ insights on the benefits and barriers of mHealth technology. Perceived benefits included improved real time monitoring, convenience, knowledge, and access to desired care. However, some participants reported concerns regarding mobile device based telehealth, with the most commonly mentioned concern being the protection of personal information (Abelson, Kaufman, Symer, Peters, Charlson, & Yeo, 2017).

Another study elicited nursing students’ opinions of telehealth systems. Respondents believed that telehealth systems eliminate some healthcare barriers; 66% would employ telehealth systems in their future careers. However, roughly one-third were hesitant to use telehealth systems due to concerns regarding the privacy and security of Protected Health Information (PHI), especially if there was “a ‘breach of the system’ and ‘personal information was left unprotected’” (Bull, Dewar, Malvey, & Szalma, 2016).

As the portability and convenience of smartphones increase, so does the risk of loss or theft (Roy, Shah, & Bhattacharya, 2016). Smartphones might be stolen for their monetary value. Smartphones can also contain a plethora of stored sensitive information, such as user names and passwords for accessing health record portals. This type of information loss can be easily prevented with the requirement of user authentication before the information in the smartphones is accessible. Interestingly, while 78% of Americans surveyed in one study stated that their smartphone data were more sensitive than data contained on their desktop computers (Holland, Hill, Rochford, Fiore, Berlowitz, & McDonald, 2013), 28% of smartphone users do not use a screen lock or other type of security measure when accessing their smartphones (Olmstead & Smith, 2017). Since users consider the data within their smartphone to be at such a high level of risk, it is quite concerning that so many do not take advantage of existing security measures in the mobile device to ensure protection of their data.

In the healthcare domain, a recent study indicated that 41% of healthcare smartphone users do not activate user authentication on their devices, even as simple as a passphrase (Cisco mConcierge, 2013). It is therefore not surprising that PHI breaches accounted for approximately 78% of all reported breaches in 2015 (Office of the National Coordinator for Health Information Technology, 2016). The potential for HIPAA (Health Insurance Portability and Accountability Act of 1996) violations is very concerning. The risk of security breaches could increase as smartphones become more widely employed to access or deliver telehealth services from anywhere and at any time. Though mHealth app based telehealth provides opportunities to narrow the healthcare access gap related to social and financial disparities, users must also consider the sensitivity of the data they exchange in smartphone apps and take proper security measures to protect highly sensitive patient data (Parati, Torlasco, Omboni, & Pellegrini, 2017). When handling sensitive information via downloaded mHealth apps on a smartphone, user authentication is a first line security measure to protect sensitive information on the smartphone.

The HIPAA security rule provides standards “to protect individuals’ electronic personal health information that is created, received, used, or maintained by a covered entity.” (Office for Civil Rights, 2017a). If clinicians are to process electronic PHI (ePHI) on a smartphone, proper security must exist to ensure that ePHI is not released to unauthorized personnel. In the next section, we present several authentication methods that can help secure sensitive data and reduce data breaches.

As a way to handle breaches that do occur, the U.S. Department of Health and Human Services (DHHS) Office for Civil Rights (OCR) created a HIPAA breach reporting tool that organizations must use to report any breaches that affect 500 or more individuals (Office for Civil Rights, 2017b). Originally created in 2009, the HIPAA breach reporting tool not only displays HIPAA breaches, but can be used to document the threats to protected health information and how reported breaches are investigated and resolved (Landi, 2017).

## GENERAL AUTHENTICATION METHODS

Authentication is the process of proving something to be true. There are three major types of information used in authentication: *what you know, what you have,* and *what you are*. The first authentication type is knowledge-based authentication in that the user knows some secret information such as a PIN, passphrase, or password (Zaidi, Shah, Kamran, Javaid, & Zhang, 2016). This authentication type requires a user to enter a PIN or password to access their smartphone or data on the smartphone prior to every use. Because of its simplicity, this authentication method has been used in many systems in recent years. Often, users choose to use a password they can remember easily to fulfill the authentication requirement. Choosing an easy or weak password makes it much less daunting for an attacker to break into the device. Attackers can take advantage of an easy password by using an attack method such as a dictionary attack (i.e., attackers use online resources to help guess the user’s password) or a brute force attack (i.e., the attacker tries all possibilities of passwords, especially if the password is short). Alternatively, the attacker can apply known information about a user (e.g., date of birth; pet’s name; favorite sport, etc.) to identify a likely password (Pfleeger, Pfleeger, & Margulies, 2015).

The second type of authentication employs something the user has, such as a token or access card (Clarke & Furnell, 2007). This authentication method is widely employed in physical building access control or online account management. Employees that work in one strictly controlled building may be assigned a RSA SecurID, which can generate an authentication code once in each time interval (e.g., one minute). An employee must use both their employee ID card and the frequently updated authentication code to obtain access to the building. Such an authentication system may use a soft token, in which the authentication code is generated by a server and sent to the user as an email or a text message. The user can then enter the code at an indicated place to get access to requested information. Though tokens are effective in providing a strong authentication method, they are always at risk of being lost or stolen (Fernandez-Aleman, Garcia, Garcia-Mateos, & Toval, 2015). A stolen or lost token may allow an unauthorized user to gain access to the confidential contents protected by the token. This could constitute a serious breach if it involves identified patient information.

If knowledge or token based authentication is not appealing to a user, biometric authentication is a third option (Zaidi, Shah, Kamran, Javaid, & Zhang, 2016). Biometric authentication uses something that is part of a user’s attributes, making it harder for an attacker to break through the authentication (Kate, Hake, Ahire, & Shelke, 2017). Two categories of biometrics used in authentication include: physiological and behavioral. Physiological biometrics is made up of a user’s unique characteristics such as fingerprints, facial characteristics, and eye patterns (Jiang & Meng, 2017). Behavior biometrics consists of behavioral traits or habits of the user, such as signature, voice, gait, and touch dynamics, which can be analyzed to determine whether it is the proper user on the device (Teh, Teoh, & Yue, 2013). Biometric authentication seems to be far superior in terms of security, as compared to other types of authentication methods, because it does not employ something the user needs to carry around or remember; instead, it is *who the user is* or *what the user has* such as the fingerprint (Laghari, Waheed-ur-Rehman, & Memon, 2016).

Though biometrics based authentication may seem appealing to some users, not all users want to utilize this form of authentication. Biometrics are unique to each individual user, thus making it a good authentication method. However, if an intruder compromises a person’s biometric authentication, it is unable to be replaced or changed, as could be easily done with a password or token (Fernandez-Aleman, Garcia, Garcia-Mateos, & Toval, 2015). Therefore, a user must consider each authentication method by comparing advantages and disadvantages of each to decide which best suits the user.

A summary of the three major types of authentication methods is presented in Table 1, wherein the benefits, disadvantages, and security power of each category are described. Further information will be provided in later sections to explain some contents in the table, such as usability issues, re-authentication, and computational power. There are also other types of authentication methods such as geo-location-based authentication and static IP based authentication.

## AUTHENTICATION METHODS ON SMARTPHONES

Knowledge-based and token-based authentication methods can be used on smartphones; they are not different from authentication on personal computers. A potentially promising authentication that may help bridge the convenience and security factors in smartphones is touch dynamics. Touch dynamics consists of analyzing and measuring each user’s unique behavior such as keystrokes for authentication purposes (Koong, Yang, & Tseng, 2014). As a benefit, the user does not have to remember or carry anything around. All that needs to be done are the user’s usual activities on the smartphone; the system will analyze the activities such as button clicks and keystrokes in the background authenticating the user. The use of touch dynamics as a security method is growing more popular recently due to the ease of utilizing this authentication function without having to add extra hardware, such as the fingerprint scanners and retinal scanners required by physiological biometrics based authentication (Jiang & Meng, 2017). On the other hand, like other previously mentioned authentication methods, there are some downsides to this method. First, smartphones have a lower computational capability than desktop computers, which may cause a delay with touch dynamic authentication use (Teh, Zhang, Teoh, & Chen, 2016). Though no additional hardware is needed, the sensors used within smartphones have an impact on the battery, requiring more frequent charging of the smartphone battery (Koong, Yang, & Tseng, 2014).

A unique feature of touch dynamics is that of re-authentication. Re-authentication can be performed continuously and transparently in the background without interfering with usability during the user’s active session (Crawford & Renaud, 2014; Shen, Yu, Yuan, Li, & Guan, 2016). The user can continue with what they are doing, while the authentication takes place in the background confirming it is the proper user. This will greatly reduce the risk of unauthorized data access by reuse of the authentication information. However, one concern about usability is whether there will be enough input for authentication to take place. For example, if a user is watching a video on his/her phone, there will be no touches from the user to use during re-authentication. In this case, perhaps the session would be inactivated or suspended until authentication takes place.

Since 2013, Apple has released multiple iPhone models wherein the user can scan their fingerprint to gain access to the smartphone. To utilize this function, the user needs to set up a ‘Touch ID’ where they identify a PIN or password and scan a finger print profile that they may use to authenticate. With this Touch ID, the user can use the fingerprint as authentication to unlock the smartphone and make purchases instead of using the PIN or password. If the device is unable to recognize the fingerprint five times in a row, the PIN or password can be used as a backup authentication to unlock the mobile device. Recently, Apple has been testing three-dimensional facial recognition for user authentication and has started to use this technology on their iPhone models introduced in September 2017. This authentication approach (Face ID) is expected to be more secure than fingerprint-based authentication (Touch ID).

## SMARTPHONE AUTHENTICATION METHODS IN HEALTH CARE

A report released in 2016 by the OCR in the DHHS stated that “PHI breaches affected over 113 million individuals in 2015.” Security breaches in smartphones could have similarly widespread impacts (Office of the National Coordinator for Health Information Technology, 2016). Without stronger security measures in smartphones in healthcare environments, especially the wide utilization of authentication methods, the breaches only stand to increase.

Currently, HIPAA does not have specific requirements for authentication on smartphones (Luxton, Kayl, & Mishkind, 2012). Because the problem of security for smartphones has the potential to grow larger, HIPAA rules may need to be updated. Smartphone specific authentication may be required to help users keep their mobile phone data secure and private. Requiring the use of authentication does not necessarily mean that one specific authentication method would be mandated. Several methods could be chosen from the available authentication methods. One option would be to utilize a token like the employee badge. Since healthcare facility employees are required to have their badges displayed during work hours that would not require additional effort. However, it would be difficult to integrate that type of authentication without additional hardware for an employee’s personal smartphone.

A second option could be to utilize touch dynamics as a background authentication. In this type of authentication, users do not have to actively authenticate each time of use. Not only will authentication happen transparently (in the background), but so will re-authentication. This type of authentication method would be quite attractive to users since it would not interrupt the workflow of healthcare services. One study reported that 90% of smartphone users would consider using this type of transparent authentication (Crawford & Renaud, 2014). This authentication type may positively affect how authentication is perceived, and could turn out to be the predominant authentication method for smartphone-based healthcare services, especially smartphone-based telehealth. For instance, smartphones contain a plethora of sensors that can capture data (e.g., physical, biological, and behavioral) (Kotz, Gunter, Kumar, & Weiner, 2016). Without the use of authentication and regular reauthentication in the telehealth practice, these sensors may capture information from a different person using the smartphone and store the information into the patient’s medical records. This could negatively impact the integrity of the medical records and misguide the decisions of clinicians (Kotz, Gunter, Kumar, & Weiner, 2016). Therefore, regular re-authentication in the background can be useful since it can identify if the smartphone’s user is the patient and restrict the unauthorized user’s access to sensors and private information contained within the smartphone.

The third option is to utilize multi-factor authentication to secure sensitive data and produce a sound authentication method based on existing technologies (Teh, Teoh, & Yue, 2013). For example, a knowledge-based and biometric-based authentication could be applied to smartphones. If an attacker breaks through the initial passcode, they will still be upheld to the fingerprint or touch dynamics authentication, making it more difficult to access the full contents of the smartphone. On the other hand, this two-factor authentication method may prove to hinder usability. Further discussion on this issue will be provided in the next section. It may be worth the risk to preserve the security of smartphones and their data, especially in the healthcare realm. Surely required authentication will not come easily, but if it is a reality, it will not be for the user’s satisfaction on usability, but rather for PHI privacy and security.

According to a Data Brief from the ONC in 2015, two factor authentication is way to satisfy the HIPAA requirement of ensuring the person gaining access to ePHI is indeed authorized to view this data (Gabriel, Charles, Henry, & Wilkins, 2015). Due to the increased security level two factor authentication provides, several healthcare institutions are beginning to utilize it, such as the Mount Sinai Hospital, Main Line Health, and WakeMed Health and Hospitals. Though this just names a few healthcare organizations utilizing two factor authentication, greater security is being sought out to secure private information from attackers and unauthorized users.

## POLICY AND MANAGEMENT ISSUES

### BRING YOUR OWN DEVICE (BYOD)

A growth in popularity of smartphones in healthcare has brought about the bring-your-own-device (BYOD) trend. This approach not only makes it more convenient for healthcare employees, but it also decreases costs for the healthcare organizations (Martinez-Perez, Torre-Diez, & Lopez-Coronado, 2015).

However, BYOD can be both beneficial and potentially detrimental at the same time. Though convenient for both the hospital and the employee, employees using their own smartphones for work can put patient PHI at a high risk of exposure to unauthorized personnel. Therefore, hospitals need to investigate how to create or enhance a policy that imposes an authentication method. Through the policy, hospitals will want to ensure that the risk of PHI exposure is identified and controlled. Perhaps hospitals that allow employees to use their own smartphones should consider having those employees register their smartphones to help ensure that the devices have proper protection equivalent to the healthcare organizations technology and security policy and standards.

### POLICY AND EDUCATION

As smartphone-based telehealth becomes more widely deployed, it will be imperative that healthcare organizations develop plans to protect the data generated in this service. It is important to recognize that unaddressed concerns regarding privacy and security could damage the success of telehealth efforts (Schwamm et al., 2017). All employees within a telehealth program should receive appropriate training in upholding privacy and security. Healthcare facilities should create a clear and concise policy of how smartphones can be used in healthcare services in a manner that promotes patient privacy and security (Ayubi, Pelletier, Sunthara, Gujral, Mittal, & Bourgeois, 2016). Since personal smartphones are allowed within healthcare facilities, the ONC provides guidance to help with the creation of a smartphone access policy.

- **Access via Smartphones:** Because smartphones are prone to many risks, such as being lost or stolen, viruses, malware, unauthorized users, and insecure networks, (Office of the National Coordinator for Health Information Technology, 2013), hospitals should consider what access via smartphone will be granted to employees. Specific to telehealth, hospitals need to ensure proper authentication, verification procedures, and encrypted data transmission (Diamantidis, 2017).
- **Restrictions/Risk Analysis:** Hospitals should consider if access via smartphone by employees needs to be restricted. The ONC suggests employing a risk analysis to determine what types of safeguards are needed to keep information secure. The risk analysis can be vital in determining whether current policies are sufficient or whether they need to be updated.
- **Risk Management Plan:** Based upon the results of the risk analysis, the ONC suggests that hospitals formulate a risk management plan that details protections and procedures to reduce risks to patient privacy and security breaches.
- **Policies and Procedures:** The next step is to develop, detail, and apply smartphone policies. The ONC suggests that these include: identifying smartphone use; determining whether hospital employees can use their own devices in the workplace, and other needed restrictions; technical controls; permissible information storage and downloads; what defines misuse; procedures for smartphone recovery and deactivation; and how security training and accountability will be instilled.
- **Ongoing Training:** Lastly, the ONC suggests ongoing training to ensure that preventable privacy breaches are avoided and to increase privacy and security awareness and safeguards (Office of the National Coordinator for Health Information Technology, 2013).

Additionally, the National Institute of Standards and Technology (NIST) provides guidance to healthcare organizations wishing to integrate the use of smartphones (Souppaya & Scarfone, 2013).

- **Smartphone Security Policy**: An organization should begin by creating a smartphone security policy, including which smartphones used by healthcare providers will be allowed to access PHI, what resources are able to be accessed, and the degree of accessibility.
- **Systems Threat Model:** Due to risks associated with smartphones, an organization should create a system threat model that helps to identify and anticipate security threats and develop solutions to potential threats.
- **General Policy**: Organizations should create a general policy, identify data communication and storage, require user and device authentication, and specify what applications can be installed and accessed.
- **Pilot Testing:** Once a smartphone solution has been identified, but prior to being finalized, the NIST suggests testing a pilot version to consider areas such as connectivity, authentication, protection, and performance.
- **Issue Secure Smartphones:** Organizational smartphones should be issued with protection prior to distribution so that there is no exposure to vulnerabilities. For instance, the University of Arizona Medical Center created a telemedicine program that utilized smartphones disbursed with password protection, HIPAA safeguards to ensure HIPAA compliance, and encryption for communication and data transmission (Zangbar et al., 2014). Additionally, each smartphone had GPS tracking so the smartphone could be relocated or remotely wiped in the event it was lost or stolen (Zangbar et al., 2014).
- **Ongoing Security Assessments:** Lastly, the NIST suggests a regular security assessment of updates, policies, and procedures to maintain a high level of protection against any threats.

A user’s training on smartphone policies will be imperative to gain patient trust and promote smartphone-based telehealth. For instance, one survey study conducted among health professionals indicated that there was poor knowledge of security issues (Ondiege & Clarke, 2017). Similarly, a survey on healthcare students showed that 82% of respondents believed that safeguards are effective in mobile devices; however, only 36% knew how to obtain such safeguards (Hewitt, Dolezel, & McLeod, 2017). An informed and educated hospital staff can amplify the trust granted by patients to an organization that engages in smartphone-based telehealth. While some patients have privacy concerns about the use of smartphone-based telehealth, trust in their healthcare provider may create a greater willingness to utilize smartphone and telehealth services (Atienza et al., 2015).

Even the most comprehensive and well-written policy will not increase patient privacy and security if hospital employees are not properly trained, nor committed to its use. A training program can ensure that each employee is aware of what is written within the smartphone policy, how it applies to their work and patients, and how to handle situations that have the potential to compromise privacy and security. Not only should employees be trained before becoming engaged in telehealth, training should be continuous to ensure current knowledge and compliance. When all employees are knowledgeable and abide by the smartphone policy, there can be a concerted, institutional effort to be cognizant of smartphone usage and to protect patient privacy and security.

Another opportunity for organizations is to implement patient training on smartphone security. A well-trained patient is not only aware of security and privacy features in their smartphone, but also knows how to use the functions of the smartphone to receive efficient telehealth care.

### USABILITY AND AUTHENTICATION

Usability plays a great role in whether users take advantage of the authentication and security features on a smartphone. Hospitals must consider whether usability is valued over the importance of protecting patient PHI on smartphones. For example, when a knowledge-based authentication is used, the problem with creating a challenging authentication lies with remembering it. Some users will choose simple and short passwords, or use one password for many accounts, or use the same passwords for a very long time, while others will write their passwords down on a piece of paper, or share their passwords with others. This makes their smartphones less secure and puts information accessible via their smartphones at risk. To make the situation even worse, a number of people (28%) chose not to use any type of passcode on their smartphones for convenience (Olmstead & Smith, 2017). One reason behind these user behaviors is that there are large numbers of online accounts each user needs to manage and it is hard to create many strong passwords and remember them. A possible solution is to use a password management program, in which the user only needs to remember one master passphrase while all other randomly generated strong passwords are encrypted with this passphrase and stored in the program (Pfleeger, Pfleeger, & Margulies, 2015).

Because usability is an important factor with stakeholders, one study sought to survey users’ opinions regarding physiological biometrics. The results indicated that respondents disliked the slow speed of authentication, the inconvenience, and the social awkwardness of using biometric authentication in public areas (Teh, Zhang, Teoh, & Chen, 2016). Users and app developers must keep in mind that surroundings and body conditions make an impact on authentication preferences (Bhagavatula, Ur, Iacovino, Kywe, Cranor, & Savvides, 2015). For example, environmental factors such as noise, lighting, and illness may have a large impact on usability of an authentication method (Koong, Yang, & Tseng, 2014).

### HUMAN ISSUES IN BIOMETRICS BASED AUTHENTICATION

Though biometrics is not something that we have to remember or carry around with us, it is something that changes. All humans are prey to the aging process; our skin will wrinkle; our hair will change color and our hand writing may get worse. Unfortunately, each authentication method has advantages and disadvantages so there is no perfect type of authentication method (Shafique et al., 2017). If biometrics as an authentication method gains popularity, there should be some type of adaption in the algorithm that is used to recognize minor changes over time but can still know the difference when an attacker is trying to penetrate the device. A similar desire applies to touch dynamics as well since some external factors, such as mood, tiredness, sickness, and distraction, may influence a user’s behavior on their smartphones (Guven & Sogukpinar, 2003).

Although authentication can increase a smartphone’s security, user convenience and usability must be taken into consideration to promote use of the secure authentication methods (Bhagavatula, Ur, Iacovino, Kywe, Cranor, & Savvides, 2015). For example, because smartphones are portable, they are accessed frequently thus making it tedious if user authentication is required for each access (Kate, Hake, Ahire, & Shelke, 2017). Because of this issue, there has been a compromise of applying a delayed authentication setting where there is a specified idle time before authentication is required (Teh, Zhang, Teoh, & Chen, 2016). Though this addresses the authentication frequency and smartphone usability, it makes the smartphone and its data less secure. Treading the line of usability and security is difficult. Though users may be offered a high level of security, they may be more reluctant to use it if it has poor usability (Teh, Zhang, Teoh, & Chen, 2016).

## CONCLUSION

In today’s world, there is a great threat to smartphone security when mobile devices are used to process highly sensitive data such as PHI in the domain of healthcare, especially in the smartphone-based telehealth services. Though some authentication methods exist to help deter attackers, each has its advantages and disadvantages. In addition, they come with usability constraints. There may therefore never be just one type of authentication that will work for every person or healthcare organization. The real struggle will be getting smartphone users to employ at least some type of authentication, even if it is not the strongest in terms of security, because doing so can still strengthen the security of PHI. To reach that goal, healthcare organizations need to put significant efforts into creating proper smartphone policy and providing training to their employees and patients.

## Figures and Tables

**Table 1:** A Summary of Three Major Types of Authentication Methods

Authentication Type	Benefits	Disadvantages	Security
Knowledge Based (e.g., PIN, password, passphrase)	Ease of use Most common authentication Applies a layer of protection	User chooses easy/weak passwords or PINs Frequent target for attackers	More susceptible to dictionary attacks and brute force attacks
User Possession (e.g., token, access card)	Without the token, unauthorized users cannot breach the device	Inconvenient Risk of damaging or losing token	Enhances security but only if token is available and not damaged
Physiological Biometric (e.g., fingerprints, facial characteristics, Iris patterns)	Simple to use Superior to other types of authentication Nothing for the user to remember or carry	Low usability deters usage Slow speed Socially awkward Potential environmental impact May require additional hardware	Increased security due to the uniqueness of physiological characteristics
Behavior Biometric (e.g., signature, voice, gait, touch dynamics)	Convenient Uses traits unique to the user to authenticate user Nothing for the user to remember or carry; no extra hardware needed Less expensive than physiological biometrics	Background re-authentication will require input Mobile devices have less computational capability Sensor usage may require more frequent charging Possible influences of external factors Requires ability to recognize user pattern changes versus attacker	Increased security. More difficult for attacker to replicate
